# Co-administration of oral killed whole-cell recombinant cholera toxin B-subunit vaccine (WC-rCTB) and live *Salmonella* Typhi Ty21a vaccine: a prospective randomized open-label trial

**DOI:** 10.1093/jtm/taag008

**Published:** 2026-02-05

**Authors:** Marianna Riekkinen, Manuela Terrinoni, Sari H Pakkanen, Joanna Kaim, Tero Vahlberg, Anna Lundgren, Anu Kantele

**Affiliations:** Meilahti Vaccine Research Center MeVac, University of Helsinki and Helsinki University Hospital, Helsinki, Finland; Department of Infectious Diseases, Inflammation Center, Helsinki University Hospital, Helsinki, Finland; Human Microbiome Research Unit, University of Helsinki, Helsinki, Finland; Department of Microbiology and Immunology, Institute of Biomedicine, University of Gothenburg, Gothenburg, Sweden; Meilahti Vaccine Research Center MeVac, University of Helsinki and Helsinki University Hospital, Helsinki, Finland; Human Microbiome Research Unit, University of Helsinki, Helsinki, Finland; Department of Microbiology and Immunology, Institute of Biomedicine, University of Gothenburg, Gothenburg, Sweden; Department of Biostatistics, University of Turku and Turku University Hospital, Turku, Finland; Department of Microbiology and Immunology, Institute of Biomedicine, University of Gothenburg, Gothenburg, Sweden; Meilahti Vaccine Research Center MeVac, University of Helsinki and Helsinki University Hospital, Helsinki, Finland; Department of Infectious Diseases, Inflammation Center, Helsinki University Hospital, Helsinki, Finland; Human Microbiome Research Unit, University of Helsinki, Helsinki, Finland

**Keywords:** Dukoral®, Vivotif®, immunogenicity, safety, antibody-secreting cells, vibriocidal antibodies

## Abstract

**Background:**

Cholera and typhoid fever are often co-endemic, making vaccine co-administration practical. However, due to lack of immunogenicity data, current guidelines advise against co-administration of the oral inactivated whole-cell recombinant cholera toxin B-subunit vaccine (WC-rCTB) and the oral live *Salmonella* Typhi Ty21a vaccine.

**Methods:**

Healthy adults (18–65 years) were randomized 1:1:1 to receive WC-rCTB with Ty21a (group Ch + Ty), WC-rCTB alone (group Ch) or Ty21a alone (group Ty). Peripheral blood mononuclear cells (PBMCs) were isolated on Days 0, 5 and 7 from all, plus on Days 12 and 14 from WC-rCTB recipients, to assess antibody-secreting cells (ASCs) specific to rCTB and to typhoidal O9,12-structures by enzyme-linked immunosorbent spot (ELISPOT) assay. Vibriocidal antibodies were assessed, and anti-rCTB IgA/IgG and anti-*S.* Typhi lipopolysaccharide (LPS) IgA/IgG/IgM were measured by enzyme-linked immunosorbent assay (ELISA) in Day 0 and 28 ± 3 serum samples. Adverse events (AEs) were recorded during one month.

**Results:**

The final study population included 63 volunteers, 21 per group. A non-significant trend towards stronger rCTB-specific ASC (IgA + IgG + IgM) peak responses was observed in group Ch + Ty compared to group Ch (geometric mean, GM 94 vs 32 ASC/10^6^ PBMC, *P =* 0.096). Serum anti-rCTB IgA and IgG fold rises (post-vaccination vs pre-vaccination) were higher in group Ch + Ty than in group Ch (IgA *P =* 0.039, IgG *P =* 0.028), whereas vibriocidal fold rises were comparable between the two groups (*P =* 0.847). ASC (IgA + IgG + IgM) peak responses to typhoidal O9,12-structures were comparable between groups Ch + Ty and Ty (GM 183 vs. 210 ASC/10^6^ PBMC, *P =* 0.684). Serum anti-*S.* Typhi LPS IgA, IgG and IgM fold rises were also similar across Ch + Ty and Ty groups (all *P*-values ≥0.145). AEs were comparable in single and co-administration groups.

**Conclusions:**

Co-administration of the oral cholera and typhoid vaccines demonstrated favourable safety and robust immunogenicity for both vaccines, supporting their simultaneous use without spacing precautions.

## Introduction

Co-administration of vaccines is generally well-tolerated and has become common practice. Since cholera and typhoid fever are largely co-endemic,^1,2^ co-administration of oral vaccines against *Vibrio cholerae* and *Salmonella* Typhi would be practical. However, data remain limited on how co-administration impacts the immunogenicity of each vaccine.

Concerns about interactions are not unfounded.^3,4^ Altered immune responses have been reported following co-admini-stration of several vaccines, including old inactivated parenteral whole-cell cholera vaccines and yellow fever vaccines,^4–6^ live oral polio and rotavirus vaccines,^3,4,7–9^ measles–mumps–rubella and varicella or yellow fever vaccines,^3,4,10–13^ Bacillus Calmette–Guérin and various other vaccines,^14^ pneumococcal conjugate and influenza or hepatitis A vaccines,^15,16^ and other conjugate vaccines with tetanus and diphtheria toxoid carriers.^17^

In general, live vaccines are routinely and safely co-administered with inactivated vaccines, including oral vaccines. The currently licensed oral typhoid vaccine contains live attenuated *S.* Typhi Ty21a bacteria in enteric-coated capsules (Vivotif®). Traveller-targeted cholera vaccines include the inactivated whole-cell plus recombinant cholera toxin B-subunit vaccine (WC-rCTB, Dukoral®) and the live attenuated CVD103-HgR cholera vaccine (Vaxchora®), both administered with bicarbonate buffer to protect antigens from gastric acid.

Despite the general safety of co-administration, specific concerns have been raised regarding the co-administration of Ty21a and bicarbonate-buffered oral cholera vaccines. Historical data support the safety and immunogenicity of a liquid Ty21a–CVD103-HgR combination^18–21^; however, Ty21a is now available only in less immunogenic enteric-coated capsules.^22^ It has been suggested that bicarbonate buffer might dissolve the capsule prematurely, exposing the live Ty21a strain to gastric acidity and thereby reducing the effective dose delivered to the small intestine. This theoretical risk has prompted caution in numerous guidelines that recommend separating administration of Ty21a and bicarbonate-buffered cholera vaccines by hours to a full day.^23–29^

To our knowledge, no studies have assessed whether bicarbonate buffer affects the immunogenicity of enteric-coated Ty21a capsules, nor the effect of co-administration with inactivated cholera vaccines. To address these gaps, we explored the immunogenicity and safety of co-administered WC-rCTB (Dukoral®) and Ty21a (Vivotif®) vaccines. Because oral vaccines elicit both systemic immune responses and intestinal immunity at the pathogen’s entry site,^30–34^ we assessed responses in both compartments: systemic (serum antibodies) and mucosal (gut-derived antibody-secreting cells, ASC^22,35^). This combined approach enabled a comprehensive assessment of humoral immunity and whether co-administration alters the responses.

## Methods

### Study design

This phase IV open-label, randomized, parallel-group trial evaluated the safety and immunogenicity of co-administered WC-rCTB and Ty21a vaccines. The study was conducted 25 October 2023–26 June 2024 at the Meilahti Vaccine Research Center MeVac, Helsinki University Hospital, University of Helsinki, Finland. Laboratory analyses were performed at the University of Helsinki (Enzyme-linked immunosorbent spot, ELISPOT) and at the University of Gothenburg, Sweden (Enzyme-linked immunosorbent assay, ELISA and vibriocidal assay).

The protocol was approved by the regional ethics committee and the Finnish Medicines Agency (EudraCT 2014-003474-16), and the study was recorded in the Clinical Trials Register (Clin.gov.trials NCT06104345). All participants provided written informed consent.

- Primary endpoints: Highest numbers of ASC specific for cholera (rCTB) or *S.* Typhi (O9,12) in peripheral blood after vaccination (peak IgA + IgG + IgM ASC/10^6^ peripheral blood mononuclear cells, PBMCs).
- Secondary endpoints: Serum anti-rCTB IgA/IgG-antibodies and anti-*S.* Typhi lipopolysaccharide (LPS) IgA/IgG/IgM- antibodies one month after vaccination (post-vaccination titres and fold rises, i.e. post-vaccination titres divided by baseline titres).

Serum vibriocidal antibodies were measured in parallel, and adverse events (AEs) were recorded for one month (not pre-specified endpoints).

### Vaccines

WC-rCTB (Dukoral®, Valneva Sweden AB, Stockholm, Sweden) contains 31.25 × 10^9^ bacteria of each of the following formalin (F)- or heat (H)-inactivated *V. cholerae* O1 strains: classical Inaba Cairo 48 (H), El Tor Inaba Phil 6973 (F) and classical Ogawa Cairo 50 (F and H) plus 1 mg rCTB in a liquid formulation. The adult regimen comprises two doses dissolved in a sodium bicarbonate buffer solution ingested 1–6 weeks apart.^29^

Each Ty21a (Vivotif®, Emergent Biosolutions, Berne, Switzerland) enteric-coated capsule contains at least 2 × 10^9^ live attenuated *S.* Typhi Ty21a bacteria, which express both typhoidal O-antigens (O9,12) but not the capsular Vi antigen. The European vaccine regimen consists of three capsules taken at 2-day intervals.^36^

### Study population and conduct

Volunteers aged 18–65 years with general good health and no previous cholera or typhoid vaccinations or episodes were randomized 1:1:1 to receive either WC-rCTB co-administered with Ty21a (group Ch + Ty), WC-rCTB alone (group Ch) or Ty21a alone (group Ty). Randomization was stratified by age (18–45/46–64 years) and sex (female/male). Travel history was recorded to evaluate possible previous exposure to *V. cholerae* and *S.* Typhi, but it did not influence eligibility. For detailed inclusion and exclusion criteria, recruitment and randomization, see Supplement 1.

Groups Ch + Ty and Ch received WC-rCTB on Days 0 and 7, and groups Ch + Ty and Ty received Ty21a on Days 0, 2 and 4. In group Ch + Ty, the first WC-rCTB and Ty21a doses were ingested simultaneously. Participants were not allowed to eat or drink for one hour before or after vaccination (fasting).

Baseline serum samples for antibody analyses and PBMCs for ASC assessments were collected before vaccination (Day 0). Based on previous studies,^22,37–39^ to catch the peak of ASC responses, PBMCs were isolated after vaccination on Days 5, 7, 12 and 14 for WC-rCTB recipients (groups Ch + Ty and Ch), and on Days 5 and 7 for group Ty. Post-vaccination serum samples were collected on Day 28 ± 3.

### Immunological analyses

To evaluate WC-rCTB responses, we assessed rCTB-specific ASC (ELISPOT), serum IgA/IgG antitoxin antibodies (ELISA) and serum vibriocidal titres.

For Ty21a, ASC specific for O9,12-LPS were enumerated using recombinant *Salmonella* Typhimurium SL2404 strain lacking Vi antigen (ELISPOT),^22^ and serum IgA/IgG/IgM were measured against *S.* Typhi LPS (ELISA).

ASC responses were analysed in all participants against all antigens, whereas vaccine-specific serum responses were assessed in all recipients of the respective vaccine plus 10 randomly selected individuals not receiving the vaccine.

#### ASC: ELISPOT

Antigen-specific ASC were detected by ELISPOT^22^ (Supplement 2). Briefly, PBMCs were incubated in antigen-coated plates (ganglioside GM1 + rCTB or SL2404) and secreted antibodies were detected by alkaline-phosphatase-conjugated anti-human IgA/IgG/IgM, followed by substrate in agarose. The spots were enumerated by a blinded researcher.

A response was defined as >3 IgA + IgG + IgM ASC/10^6^ PBMC (limit of detection, LOD^40^). We report peak responses defined as the highest number of ASC/10^6^ PBMCs measured on Day 5, 7, 12 or 14 for rCTB, and on Day 5 or 7 for SL2404.

#### Serum antibodies: ELISA

For serum antibody ELISA^41^ (Supplement 2), plates were coated with GM1 + rCTB or *S.* Typhi LPS. Briefly, serum samples were serially diluted in the plates, and endpoint antibody titres were determined by addition of horse-radish peroxidase-conjugated anti-human IgA/IgG/IgM, followed by ortho-phenylenediamine. Plates were read at 450 nm (rCTB) or 490 nm (LPS).

Seroconversion was defined as ≥2-fold titre increase (fold-rise) from baseline to Day 28 ± 3.

#### Vibriocidal assay

Serum vibriocidal activity was measured against *V. cholerae* O1 El Tor Inaba^42^ (Supplement 2). Briefly, serum samples were serially diluted and mixed with cholera bacteria and guinea pig complement. After incubation, bacterial growth was assessed by measuring optical density at 600 nm.

Vibriocidal seroconversion was defined as ≥4-fold titre increase (fold-rise) from baseline to Day 28 ± 3.

### Safety

AEs were monitored at each visit via a structured interview. Solicited and unsolicited AEs were recorded at Visits 2–5, and only unsolicited ones at Visit 1 and the final visit. Solicited AEs comprised nausea, vomiting, abdominal pain, loose stools, diarrhoea, constipation, axillary temperature >37.5°C, headache, muscle/joint pain, rash, fatigue and vertigo. Severity (mild, moderate, severe) was self-assessed (Supplement 5). Causality was evaluated by the study physician as probable, possible or unlikely. AEs evaluated as probably/possibly vaccine-related were followed until resolution. Serious AEs (SAEs) were to be reported 6 months post-vaccination.

### Statistical analysis

Sample size was based on previous studies of ASC responses to oral vaccines.^22,34,37–40,43–45^ Zero ASC values were replaced by one to allow geometric mean (GM) with 95% confidence interval (CI) calculations. The Mann–Whitney *U* and the Kruskal–Wallis tests were used for group comparisons for the non-normally distributed continuous variables. Fisher’s exact test was used to evaluate responder frequencies, AE rates and demographic binary variables. Two-tailed *P*-values <0.05 were considered statistically significant. Analyses were performed by IBM SPSS Statistics for Windows (version 29) and GraphPadPrism (version 10.4.1.).

## Results

### Participants and study conduct

**Figure 1:**
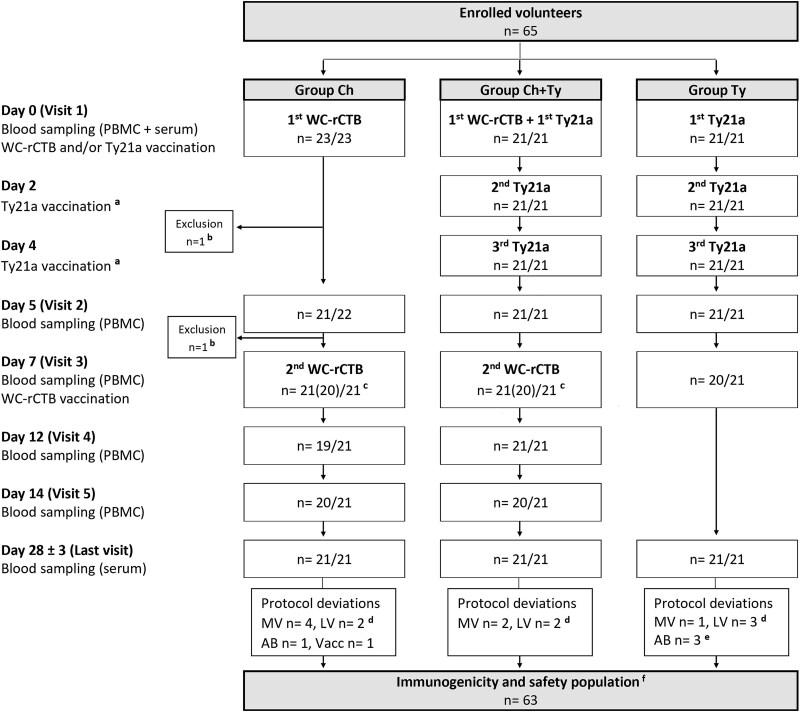
Flow chart of open-label, randomized, parallel-group study evaluating the safety and immunogenicity of co-administered oral killed whole-cell recombinant cholera toxin B-subunit vaccine (WC-rCTB) and live *S.* Typhi Ty21a vaccine (Ty21a). Adverse events (AEs) were followed throughout the study (Visit 1–Last Visit), and serious AEs were to be reported up to 6 months after the last vaccination. PBMCs, peripheral blood mononuclear cells; MV, missed visit; LV, last visit out of protocol window; AB, antibiotic treatment during the study; Vacc, parenteral hepatitis A + B vaccination on Day 21; ^a^Self-administration of Ty21a vaccine was ensured by text message confirmation. ^b^Reason for exclusion: loss to follow-up/personal matters. ^c^Number of vaccinated participants is 21/21 in all groups. One participant in group Ch and one in group Ch + Ty missed Visit 3 due to mild respiratory symptoms but received the second dose of WC-rCTB at home on Day 7.^d^ Seven last visits (Day 28 ± 3) were out of protocol window: two (2/21) in group Ch (Days 20 and 23), two (2/21) in group Ch + Ty (Days 22 and 35) and three (3/21) in group Ty (Days 22, 32 and 37). ^e^No AB during the Ty21a vaccination period (Days 0–7). ^f^Of the 63 participants included in the immunogenicity and safety analyses, 56/63 attended all planned visits, six (6/63) missed one and one (1/63) missed two visits.

Of the 65 randomized volunteers, 63 (21/group) completed the study and comprised the immunogenicity and safety population (Figure 1). The groups were balanced with respect to age, sex, body mass index, ethnicity and travel history (all *P*-values ≥0.767, Table 1). None of the pre-existing medical conditions or regular medications were thought to affect vaccine responses (Table 1, Supplement 3).

**Table 1:** Baseline characteristics of study population included in safety and immunogenicity analyses (*n* = 63)

	All *n* = 63	Group Ch *n* = 21	Group Ch + Ty *n* = 21	Group Ty *n* = 21	*P*-value^a^
Median age, years (IQR)	34 (20)	31 (18.5)	34 (22.5)	36 (24)	0.968
Male, *n* (%)	26 (41.3)	9 (42.9)	9 (42.9)	8 (38.1)	1.000
Median BMI (IQR)	24.1 (5.9)	24 (6.3)	25.4 (5.9)	23.7 (5.5)	0.827
Caucasian ethnicity, *n* (%)	60 (95.2)	20 (95.2)	20 (95.2)	20 (95.2)	1.000
Lived in LMIC >3 months^**b**^, *n* (%)	3 (4.8)	0 (0)	2 (9.5)	1 (4.8)	0.767
Travelled to LMIC during past 3 years^**c**^, *n* (%)	14 (22.2)	4 (19)	5 (23.8)	5 (23.8)	1.000
Pre-existing medical condition^**d**^, *n* (%)	34 (54)	11 (52.4)	9 (42.9)	14 (66.7)	0.339
Neurologic	10 (15.9)	5 (23.8)	3 (14.3)	2 (9.5)	0.575
Psychiatric	10 (15.9)	2 (9.5)	2 (9.5)	6 (28.6)	0.188
Cardiometabolic	9 (14.2)	2 (9.5)	3 (14.3)	4 (19)	0.901
Respiratory	8 (12.7)	1 (4.8)	3 (14.3)	4 (19)	0.508
Dermatologic	8 (12.7)	2 (9.5)	0 (0)	6 (28.6)	0.022
Gastrointestinal	5 (7.9)	2 (9.5)	2 (9.5)	1 (4.8)	1.000
Other	8 (12.7)	3 (14.3)	2 (9.5)	3 (14.3)	1.000
Regular systemic or inhaled medication^**d**^, *n* (%)	24 (38.1)	5 (23.8)	7 (33.3)	12 (57.1)	0.097

Volunteers received either oral killed whole-cell recombinant cholera toxin B-subunit vaccine (Group Ch), live *S. Typhi* Ty21a vaccine (Group Ty) or both (Group Ch + Ty, first doses ingested simultaneously).

IQR, interquartile range; BMI, body mass Index [weight in kilograms/(height in meters)^2^]; LMIC, low- or middle-income country (according to World Bank income groups 2023).

^a^ *P*-values are calculated with Kruskal–Wallis for age and BMI and with Fisher’s exact test for the binary variables.

^b^Duration of stay 3.5–33 years, all three participants had left the LMIC >7 years ago.

^c^Duration of stay 1 week–1 month, two participants (2/14) had returned from the LMIC <4 months ago.

^d^None of the pre-existing medical conditions or regular medications were thought to affect safety or vaccine responses. See Supplement 3 for details.

A total of 56/63 participants attended all planned visits (Figure 1). All vaccine doses were ingested on the intended days. Participants with protocol deviations were included in safety and immunogenicity analyses (Figure 1).

### Immunogenicity

#### Responses to WC-rCTB vaccine

##### rCTB-specific ASC

**Figure 2:**
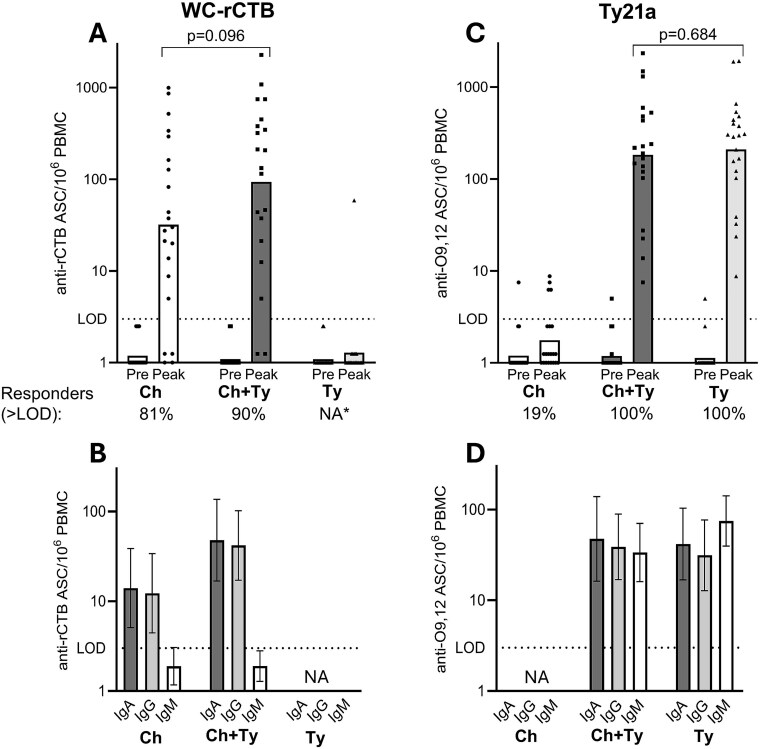
Antibody-secreting cell (ASC) responses to cholera (A, B) and *S.* Typhi antigens (C, D) in participants receiving either oral killed whole-cell recombinant cholera toxin B-subunit (WC-rCTB) vaccine (Group Ch, *n* = 21), live *Salmonella* Typhi Ty21a (Ty21a) vaccine (Group Ty, *n* = 21) or both (Group Ch + Ty, *n* = 21, first doses ingested simultaneously). (A) and (C) present antigen-specific IgA + IgG + IgM ASCs before (Pre, Day 0) and after (Peak) vaccination. The Peak response to rCTB (A) is the highest of Day 5, 7, 12 or 14 values and to O9,12 (B) the highest of Day 5 or 7 values. (B) and (D) present the immunoglobulin isotype distribution of respective ASC peak responses. Individual ASC responses are represented as symbols, geometric means as bars and 95% confidence intervals as error bars. The proportions (%) of responders of all evaluable participants are presented below (A) and (C) (one participant in Group Ch + Ty not evaluable for anti-rCTB and one for anti-O9,12). Mann–Whitney *U* test was used to calculate exact *P*-values. rCTB, recombinant cholera toxin B-subunit; O9,12, typhoid O9- and O12-lipopolysaccharides; PBMCs, peripheral blood mononuclear cells; LOD, limit of detection ^*^Responder rates are not calculated, since no Day 12 or 14 samples are available from Group Ty.

Before vaccination, none of the 62 participants with available baseline values had rCTB-specific ASC above LOD (>3/10^6^ PBMC) (Figure 2A).

In group Ch + Ty, 18/20 (90%; one participant with missing rCTB-specific ASC values due to a technical error was not evaluable) and in group Ch, 17/21 (81%) volunteers responded to WC-rCTB with rCTB-specific ASC > LOD (*P =* 0.663). The GM peak rCTB-specific response (ASC/10^6^ PBMC) was 94 (95% CI 34–257) in group Ch + Ty compared to 32 (95% CI 12–88) in group Ch (*P =* 0.096, Figure 2A). In both groups receiving WC-rCTB, the rCTB-specific ASC response was dominated by IgA- and/or IgG-ASC, with very few IgM-ASC (Figure 2B).

In group Ty, only one participant responded to rCTB (peak 59 ASC/10^6^ PBMC on Day 7) (Figure 2A). This was interpreted to reflect prior exposure to enteric pathogens, potentially including enterotoxigenic *Escherichia coli* (ETEC) expressing heat-labile toxin (LT), which is highly homologous to cholera toxin,^46^ on a trip to a low- and middle-income country (LMIC) one week before vaccination. A concomitant ASC response was seen against whole inactivated ETEC and enteroaggregative *E. coli* in this participant, a pattern not observed in the other vaccinees (Riekkinen *et al.*, manuscript in preparation).

##### Serum anti-rCTB and vibriocidal antibodies

**Figure 3:**
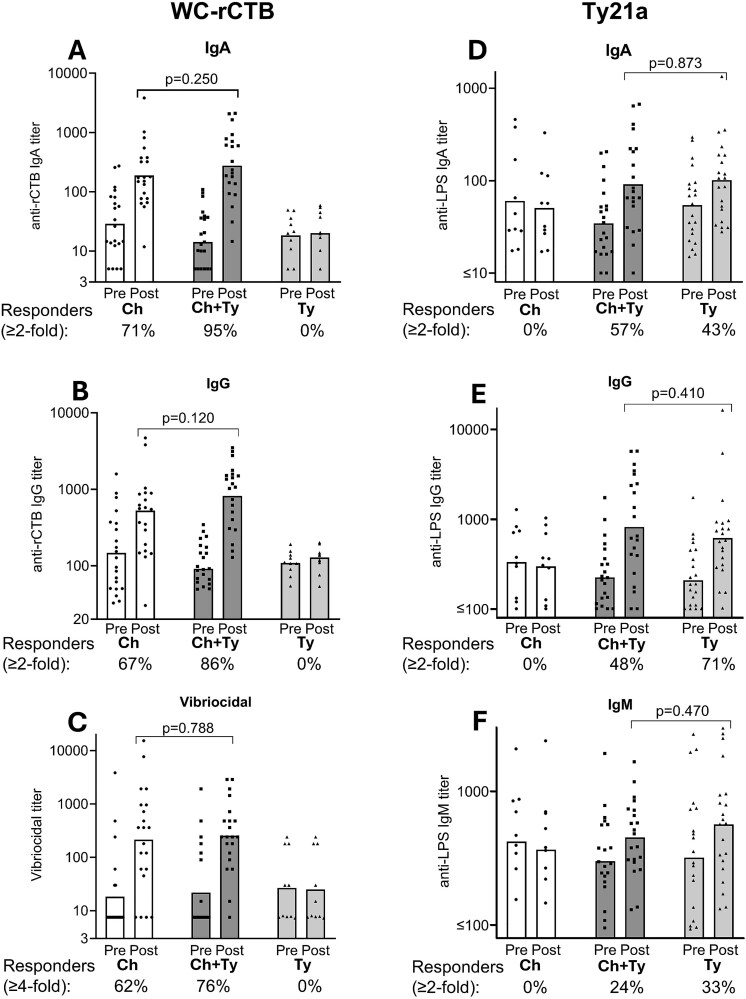
Serum antibody responses to cholera (A–C) and *S.* Typhi antigens (D–F) in participants receiving either oral killed whole-cell recombinant cholera toxin B-subunit (WC-rCTB) vaccine (Group Ch, *n* = 21), live *Salmonella* Typhi Ty21a (Ty21a) vaccine (Group Ty, *n* = 21) or both (Group Ch + Ty, *n* = 21, first doses ingested simultaneously). Serum responses were assessed in all recipients of the respective vaccine plus 10 randomly selected individuals not receiving the vaccine (*n* = 10 for Group Ty in (A–C) and for Group Ch in (D–F). Individual titres are represented as symbols and geometric means as bars. The proportions (%) of responders in each group are presented below each panel. Mann–Whitney *U* test was used to calculate exact *P*-values. Pre, pre-vaccination (Day 0); Post, post-vaccination (Day 28 ± 3); rCTB, recombinant cholera toxin B-subunit; LPS, lipopolysaccharides of *S.* Typhi.

Most participants had detectable, yet low anti-rCTB IgA and IgG prevaccination titres (Figure 3A and B). Seroconversion rates (≥2-fold increase) in groups Ch + Ty and Ch were 20/21 (95%) vs 16/21 (76%) (*P =* 0.184) for IgA and/or IgG, 20/21 (95%) vs 15/21 (71%) for IgA (*P =* 0.093) and 18/21 (86%) vs 14/21 (67%) for IgG (*P =* 0.277), respectively. Although anti-rCTB post-vaccination titres did not differ in groups receiving WC-rCTB (IgA *P =* 0.250, IgG *P =* 0.120, Figure 3A and B), the GM fold rises were ~2-fold higher in the co-administration group (IgA *P =* 0.039, IgG *P =* 0.028, Table 2). This may partly be due to slightly, but not statistically significantly, lower prevaccination titres in group Ch + Ty than in group Ch (*P =* 0.189 for IgA and *P =* 0.618 for IgG).

**Table 2:** Magnitudes of serum antibody responses (fold rises) to cholera and *S.* Typhi antigens

	Fold rises^a^ (GM, 95% CI)			
	Group Ch	Group Ch + Ty	Group Ty	*P*-value^b^
**Responses to WC-rCTB**	*n* = 21/21	*n* = 21/21	*n* = 10/21	Ch vs Ch + Ty
Anti-rCTB IgA	6.6 (3.3–13.3)	16.0 (9.0–28.7)	1.1 (0.9–1.3)	0.039
Anti-rCTB IgG	3.6 (2.0–6.3)	7.6 (4.6–12.5)	1.1 (0.9–1.3)	0.028
Vibriocidal	11.7 (4.3–31.6)	11.7 (5.6–24.6)	0.9 (0.80–1.1)	0.847
**Responses to Ty21a**	*n* = 10/21	*n* = 21/21	*n* = 21/21	Ty vs Ch + Ty
Anti-LPS IgA	0.9 (0.6–1.1)	2.7 (1.9–3.7)	1.9 (1.5–2.2)	0.145
Anti-LPS IgG	0.9 (0.7–1.1)	3.6 (2.1–6.3)	3.0 (2.1–4.1)	0.916
Anti-LPS IgM	0.9 (0.7–1.1)	1.5 (1.2–2.0)	1.8 (1.4–2.3)	0.150

Volunteers received either oral killed whole-cell recombinant cholera toxin B-subunit (WC-rCTB) vaccine (Group Ch), live *S.* Typhi Ty21a (Ty21a) vaccine (Group Ty) or both (Group Ch + Ty, first doses ingested simultaneously).

GM, geometric mean; CI, confidence interval; rCTB, recombinant cholera toxin B-subunit; LPS, lipopolysaccharides of *S.* Typhi.

^a^Fold rises are calculated as the titre in samples collected one month after vaccination (Day 28 ± 3) divided by the titre in samples collected before vaccination (Day 0).

^b^Mann–Whitney *U* test was used to calculate exact *P*-values.

A minority of vaccinees had detectable vibriocidal prevaccination titres (Figure 3C). The vibriocidal seroconversion rate (≥4-fold increase) was 16/21 (76%) in group Ch + Ty and 13/21 (62%) in group Ch (*P =* 0.506). The post-vaccination vibriocidal titres (*P =* 0.788, Figure 3C) and fold rises (*P =* 0.847, Table 2) did not differ between the two groups.

No serum antitoxin or vibriocidal responses were seen in group Ty (Figure 3A–C, Table 2).

#### Responses to Ty21a vaccine

##### O9,12-specific ASC

Before vaccination, 3/63 participants—one in each group—had detectable but low numbers of O9,12-specific ASC (≤7.5 ASC/10^6^ PBMC) (Figure 2C).

After vaccination, all sampled Ty21a recipients (20 in group Ch + Ty and 21 in group Ty) developed O9,12-specific ASC > LOD (Figure 2C). The responses did not differ between group Ch + Ty (GM 183 ASC/10^6^ PBMC, 95% CI 89–375) and group Ty (GM 210, 95% CI 114–389) (*P =* 0.684, Figure 2C). Strong O9,12-specific responses were observed in all three immunoglobulin isotypes (IgA, IgG and IgM) in groups Ch + Ty and Ty (Figure 2D).

In group Ch, 4/21 (19%), participants had few O9,12-specific ASC (≤10/10^6^ PBMC) after vaccination and the rest were non-responders (Figure 2C).

##### Serum anti-*S.* Typhi LPS antibodies

Most participants had detectable anti-*S.* Typhi LPS IgA, IgG and IgM antibodies before vaccination (Figure 3D–F). Responder frequencies (≥2-fold increase) in groups Ch + Ty and Ty were comparable 14/21 (67%) for any Ig isotype (IgA/IgG/IgM) (*P =* 1.000), 12/21 (57%) vs 9/21 (43%) for IgA (*P =* 0.538), 10/21 (48%) vs 15/21 (71%) for IgG (*P =* 0.208) and 5/21 (24%) vs. 7/21 (33%) for IgM (*P =* 0.734), respectively. No differences were observed between the two groups in anti-*S.* Typhi LPS pre- or post-vaccination titres or fold rises (Figure 3D–F, Table 2, *P* > 0.05).

In group Ch, no anti-*S.* Typhi LPS antibody responses were elicited (Figure 3D–F, Table 2).

### Safety

During the 1-month follow-up, 47/63 (75%) participants reported 108 solicited and 51 unsolicited AE episodes considered as probably or possibly related to study vaccines. Most episodes were mild, with fewer moderate (27/159, 17%) or severe (2/159, 1%). Nearly all occurred before Day 15; a single case of mild reactive monoarthritis in group Ty was observed on Day 24 in the participant with a recent LMIC travel. No SAEs were reported, and co-administration did not increase AE incidence rate or severity (Supplements 4 and 5).

In addition, 18/63 (29%) participants reported 21 AE episodes considered unlikely related to study vaccines (Supplement 6).

## Discussion

### Rationale and main findings

Concerns about buffer interference have led to recommendations for staggered administration of oral bicarbonate-buffered cholera vaccines and enteric-coated Ty21a capsules.^23–29^ In our study, co-administration of first doses proved well tolerated, and robust systemic and mucosal immune responses to both vaccine regimens were observed. A signal of enhanced cholera antitoxin-specific responses was observed but requires further confirmation.

### Relevance of co-administration

Cholera vaccination is recommended for travellers to cholera-endemic countries if they have a high risk of exposure (for example, humanitarian aid workers).^23,25–27,32^ However, WC-rCTB may also provide some short-term cross-protection against a major cause of travellers’ diarrhoea, LT-producing ETEC^47–51^ due to homology between cholera toxin and LT B-subunits.^46^ Although clinical protection against ETEC is limited,^47,48^ some countries have authorized WC-rCTB for prevention of ETEC travellers’ diarrhoea in special circumstances and in particularly vulnerable individuals, such as travellers with increased susceptibility to travellers’ diarrhoea or increased risk of severe or complicated disease.^52^

Typhoid vaccination is recommended for travellers to endemic regions, particularly to South Asia.^24,26,28,33^ The oral live Ty21a offers some practical and immunological benefits over the Vi-based parenteral typhoid vaccines: it is easy to administer and induces mucosal immune responses at the intestinal pathogen’s entry site.^22,33,34,37,38,40,43^ Ty21a has been shown to elicit cross-reactive immune responses against paratyphoid A and B^43,53^ and some other *Salmonella* serovars.^40^ Cross-protective efficacy has been demonstrated against *Salmonella* Paratyphi B.^54^ Ty21a may also exert some non-antigen-specific effects on both innate and adaptive immunity, as reported with oral live vaccines.^55^

Thus, while waiting for licensed vaccines against ETEC, paratyphoid and non-typhoidal *Salmonellas*, there is a theoretical rationale for co-administering WC-rCTB and Ty21a to selected high-risk travellers. Co-administration could extend protection beyond cholera and typhoid fever to include some co-endemic pathogens relevant to travellers.

### Safety

Although not powered for safety outcomes, our findings align with the established safety profiles of both vaccines.^56–58^ The mild reactive monoarthritis in one participant resolved without sequelae and was probably related to exposures during recent LMIC travel, as evidenced by ASC responses to additional enteric pathogens. The relatively high frequencies of mild AEs likely reflect the use of detailed symptom interviews, similar to findings in previous trials.^16,59^ Importantly, no increase in AE frequency or severity was observed in the co-administration group.

### Immunogenicity

#### Mucosal and systemic immune responses

Intestinal immune responses were assessed by quantifying vaccine antigen-specific ASC, which—after initial activation by antigen encounter at the intestinal mucosa—appear transiently in the peripheral blood before homing back to the gut.^22,35,39^ ELISPOT detects these ASCs at a single-cell level, allowing sensitive measurement of mucosal vaccine responses, with no baseline background. Combined with serum antibody assays reflecting systemic immune responses, these approaches provide a comprehensive view of vaccine-induced humoral immunity.

#### Immune response to WC-rCTB vaccine

WC-rCTB induces both systemic (serum vibriocidal and antitoxin antibodies) and mucosal (intestinal IgA antibodies) responses.^31,32^ While definitive mechanistic correlates of protection for oral cholera vaccines have not been established, vibriocidal antibody titres are the most used immunogenicity measure in cholera vaccine trials.^31,32,60^

Importantly, co-administration did not reduce the immunogenicity of the WC-rCTB vaccine: no significant differences were observed in either vibriocidal or anti-rCTB seroconversion rates or post-vaccination titres/ASC between groups receiving WC-rCTB.

In fact, we observed a trend towards enhanced antitoxin responses in the co-administration group, with significantly higher serum anti-rCTB IgA and IgG fold rises and a tendency for higher responder frequencies. This may partly reflect slightly lower baseline titres in the co-administration group, yet a similar trend for enhancement was observed in rCTB-specific ASC without baseline confounding. As for the vibriocidal responses, both seroconversion and response magnitudes (post-vaccination titres and fold rises) were comparable between groups.

Possible mechanisms for enhancement include the activation of innate immune pathways by the live-attenuated Ty21a vaccine, facilitating general antigen uptake and presentation, and promoting T- and B-cell activation or recruitment.^55^ Further studies are needed to clarify these mechanisms and their impact on co-administered antigens.

Consistent with earlier findings,^44,45^ the rCTB-specific ASC response—typical for a T-cell-dependent protein antigen—consisted mainly of IgA and IgG ASC, with minimal IgM ASC.

#### Immune response to Ty21a vaccine

Protection afforded by Ty21a correlates both with serum IgG and gut-derived IgA ASC responses to *S.* Typhi LPS.^22,33^ In our study ASC responses to typhoidal O9,12-antigens and LPS-specific IgA, IgG and IgM serum antibodies were comparable between the co-administration and the Ty21a-only groups. High baseline anti-LPS serum antibody levels, probably reflecting cross-reactivity, may explain the modest fold rises and responder frequencies. However, ASC responses were strong, with 100% responders in both groups receiving Ty21a.

As shown before, ASC responses to Ty21a consisted of not only IgA and IgG but also IgM ACS in substantial numbers,^22,34,43^ a finding likely related to the polysaccharide nature of the O9,12 antigens.

### Implications for clinical practice

Our data demonstrate that the first Ty21a capsule (Vivotif®) can be co-administered with the bicarbonate-buffered WC-rCTB (Dukoral®) at a single pretravel visit—without compromising immunogenicity or safety. Together with previous trials showing successful co-administration of liquid Ty21a and CVD103-HgR,^19–22^ our study supports reconsideration of recommendations that impose intervals between Ty21a and oral cholera vaccines. For CVD103-HgR (Vaxchora®), this should be confirmed in dedicated studies.

### Limitations

Our findings focus on travellers, and results may not be generalizable to endemic settings, where pre-existing intestinal immunity may act against the vaccine antigens, preventing effective induction of the mucosal immune response.^34,61,62^ Furthermore, in endemic settings, the use of WC-rCTB and Ty21a vaccines is minimal and, instead, non-buffered bivalent (O1, O139) whole-cell-only cholera vaccines and injectable typhoid conjugate vaccines are used for mass vaccinations promoted by GAVI, the Vaccine Alliance.^63,64^

Cell-mediated responses were not assessed. For cholera, these mainly support antibody production,^31,32^ making a separate evaluation less relevant. While T cells may contribute more directly to typhoid protection,^33,65^ co-administration-related selective interference with only cell-mediated responses is unlikely, and humoral responses are considered to provide a sufficient measure of co-administration effects.

## Conclusion

Co-administration of WC-rCTB and Ty21a neither impaired mucosal or systemic responses to either vaccine nor compromised safety. These findings support administering both vaccines together to travellers and argue against spacing recommendations.

## Supplementary Material

taag008_CholeraTyphoid_Supplementary_files

## Data Availability

Data can be made available by the corresponding author upon reasonable request, provided that any applicable privacy and ethical considerations are met.

## Presentation of preliminary data

Vaccines for Enteric Diseases meeting, 24–26 June 2025, Gothenburg, Sweden (by M.R.).
