# Supplementary material for: Co-administration of oral killed whole-cell recombinant cholera toxin B-subunit vaccine (WC-rCTB) and live Salmonella Typhi Ty21a vaccine: a prospective randomized open-label trial
Source: J Travel Med. 2026 Feb 5;33(2):taag008. doi: 10.1093/jtm/taag008 (PMC13017230; doi:10.1093/jtm/taag008)
Supplement: taag008_CholeraTyphoid_Supplementary_files [file taag008_choleratyphoid_supplementary_files.docx]

**SUPPLEMENTARY FILES**

**Supplementary file 1.** Inclusion and exclusion criteria. Recruitment and randomization details.

**Inclusion criteria**

1. Male or female subjects aged ≥18 to ≤65 years.

2. General good health as established by medical history and physical examination.

3. Written informed consent.

4. Females of childbearing potential must agree to use an efficacious hormonal or barrier method of birth control during the study (14 days before immunization to Day 28±3). Abstinence is acceptable.

5. Available for all visits scheduled in this study.

**Exclusion criteria**

1. Prior vaccination against typhoid fever or cholera.

2. History of clinical typhoid fever or cholera.

3. Immunization with any other vaccine (oral or parenteral) within 4 weeks prior to study period or vaccination planned during it.

4. Current intake of antibiotics or end of antibiotic therapy <8 days before first IMP administration.

5. Chronic (longer than 14 days) administration of immunosuppressants or other immune modifying drugs within 6 months before the first dose of IMP; oral corticosteroids in dosages of ≥0.5 mg/kg/d prednisolone or equivalent are excluded; inhaled or topical steroids allowed.

6. Acute or chronic clinically significant gastrointestinal disease.

7. Any confirmed or suspected immunosuppressive or immunodeficient condition, including human immunodeficiency virus (HIV) infection.

8. Pregnancy or lactation.

9. Acute disease at the time of enrolment (defined as the presence of a moderate or severe illness with or without fever (fever defined as body temperature of ≥38 °C).

10. Alcohol or drug abuse.

11. Suspected non-compliance.

12. Use of any investigational drug or vaccine within 30 days preceding the first dose of study vaccine, or planned use during the study period.

13. Any clinically significant history of known or suspected anaphylaxis or hypersensitivity reaction based on the judgement of the investigator.

14. Employee at the investigational site or relative or spouse of the investigator.

15. Any other criteria which, in the investigator’s opinion, would compromise the ability of a subject to participate in the study, a subject’s well-being, or the outcome of the study.

Living outside of Finland for more than three months at any point in life and any travel outside of Europe or North America during the past three years was documented, but did not influence eligibility.

**Recruitment**

Volunteers were recruited by sending email advertisements to students and personnel working on Meilahti Hospital and Helsinki University Campuses. A recruitment announcement was visible on the public web page of Meilahti Vaccine Research Center and the internal web page of Helsinki University Hospital (HUH).

**Randomization**

Randomization at a 1:1:1 ratio to the three treatment groups was done by the electronic Case Report File System (eCRF) of HUH. Randomization was stratified by age (18-45/46-64 years) and sex (female/male), and the size of each randomization block was 6.

**Supplementary file 2.** Methodology in detail

**ELISPOT antigens**

Microplates (F96 Cert Maxisorp Nunc, Thermo Scientific) were coated with 5µg/ml (50µl/well) Ganglioside GM1 Ovine Brain (Avanti) for 2h at 37^o^C followed by 2.5µg/ml (50µl/well) recombinant cholera toxin B-subunit (rCTB, Valneva, Sweden) overnight at ambient temperature. The plates were washed before adding rCTB.

Recombinant *Salmonella* Typhimurium bacteria expressing typhoidal O9- and O12- antigens but no Vi-antigen (strain SL2404) were cultured on nutrient agar plates to determine their concentration in the suspension, and formalin-killed as described previously [1, 2]. The inactivated bacterial suspension was stored at -20^o^C, thawed and adjusted to 10^9^ bacteria/ml in phosphate buffered saline (PBS tablets pH7.4, Medicago) for coating (50µl/well) overnight at ambient temperature.

The antigen-coated microplates were stored at -20^o^C for a maximum of two weeks before use.

**Isolation of peripheral blood mononuclear cells**

Peripheral blood mononuclear cells (PBMC) were isolated from fresh heparinized venous blood by Ficoll-Paque (Cytiva) density-gradient centrifugation [1], washed twice in Hank’s buffered salt solution (Gibco) and adjusted to 2 x 10^6^ cells/ml in RPMI-1640 medium (Sigma-Aldrich) supplemented with 10% fetal bovine serum (Gibco), penicillin (100 µg/mL), streptomycin (100 µg/mL), and 3 µg/mL L-glutamine (2 mM) (Penicillin-Streptomycine-Glutamine 100X, Gibco), as previously described [1].

**ELISPOT**

After thawing, the antigen-coated microplates were blocked with 1% bovine serum albumin (BSA, Biowest) in PBS (30min, 37^o^C). Freshly isolated PBMC were added (2 x 10^6^ cells/ml, 50 µL/well, total 2.4 × 10^6^ cells/antigen and 8 × 10^5^ cells/Ig isotype) and allowed to secrete antibodies for 2–3 hours at 37^o^C. The antigen-bound antibodies were detected by adding alkaline-phosphatase-conjugated goat anti-human IgA, IgG (Sigma-Aldrich) or IgM (SouthernBiotech) diluted in 1% BSA-PBS (IgA 1:30 000, IgG: 1:500, IgA 1:5000) (overnight, 4°C). The plates were washed between every step. The alkaline phosphatase substrate 5-bromo-4-chloro-3-indolyl phosphate p-toluidine salt (Sigma–Aldrich) dissolved in a 2-amino-2-methyl-l-propanol buffer pH 10.25 (2-amino-2-methyl-l-propanol 99%,Thermo scientific; Magnesium chloride hexahydrate, Sigma-Aldrich; Triton X-100, Sigma-Aldrich) was applied in melted agarose according to previously described principles [1] resulting in the formation of spots. The spots – each representing one ASC – were enumerated manually under a low magnification microscope.

**ELISA**

Serum IgA and IgG antibodies against rCTB and IgA, IgM and IgG antibodies against *S*. typhi lipopolysaccharides (LPS) were measured by ELISA, according to previously described principles [3, 4]. First, 96-well microplates (269620, Nunc Thermo Scientific) were coated overnight with 0.5µg/ml GM1 (L. Svennerholm, University of Gothenburg, in-house preparation) or with 1 µg/ml *S*. typhi LPS (Sigma-Aldrich). On the next day, GM1 coated plates were incubated with 0.5µg/ml rCTB (Valneva, Sweden) for 60 min. The plates were blocked with BSA, and prediluted serum samples (1:10 for IgA and IgM, 1:30 for IgG) were added and further serially diluted in the plates (3-fold, 7 additional steps). After 90 minutes, horse-radish peroxidase-conjugated rabbit anti-human IgM, IgA or IgG (Jackson ImmunoResearch Laboratories, USA) was added. After another 90 min, plates were developed with ortho-phenylenediamine (1mg/ml) in sodium citrate buffer (0.1M) with H_2_O_2_ added directly before development. All incubations were performed at ambient temperature and plates were washed between steps. Finally, after 20 min, plates were directly read at 450 nm (rCTB) or at 490 nm (LPS) after addition of 1M sulphuric acid to stop the reaction. Endpoint titers were determined as the reciprocal dilutions giving an absorbance of 0.4 above background using BioTek Gen5.11 software and 4-parameter logistic regression for creation of titration curves. For statistical evaluations, IgA titers below the initial dilution were assigned a value of 5.

**Vibriocidal assay**

Serum vibriocidal activity was assessed according to previously described principles [5]. *V. cholerae* O1 bacteria (serotype Inaba, strain T19479) were cultivated on Luria Bertani (LB) agar overnight at 37°C and then in LB broth (37°C, shaking) to an OD600nm of 0.6–1.0. The culture was diluted 1:50 in saline to ∼2×10^7^ bacteria/ml. Serum samples were heat-inactivated (56^◦^C, 30 min), prediluted 1:10 in saline in the first well of the plate and then further serially 2-fold diluted in the same buffer in the plate (10 additional steps, final volume 50 ul per well). Next, 50 µl of prediluted guinea pig complement (1:12, Biojet Service, Sweden) and 50 μl of diluted *V. cholerae* suspension were added to the serum containing wells (final serum dilution 1:30 in the first well). After incubation (37°C, 1 h, shaking), 50 µl of 4-times concentrated LB broth was added giving a final volume of 200μl/ in each well. Plates were further incubated (37°C, 2 h, no shaking), and OD600 was measured. Negative controls (without serum and/or complement) and a reference serum were included. The vibriocidal titer was defined as the reciprocal of the highest dilution showing ≥50% reduction in optical density compared to that of control wells without serum. Samples with no growth inhibition were assigned a titer of 7.5.

**References in supplement 2** [in brackets the reference number used in the actual manuscript, if applicable]:

1 Kantele A. Antibody-secreting cells in the evaluation of the immunogenicity of an oral vaccine. Vaccine 1990; 8(4):321–6. [22]

2 Pakkanen SH, Kantele JM, Kantele A. Cross-reactive gut-directed immune response against Salmonella enterica serovar Paratyphi A and B in typhoid fever and after oral Ty21a typhoid vaccination. Vaccine 2012; 30(42):6047–53. [43]

3 Jertborn M, Åhrén C, Holmgren J, Svennerholm AM. Safety and immunogenicity of an oral inactivated enterotoxigenic Escherichia coli vaccine. Vaccine 1998; 16(2):255–60. [41]

4 Lundgren A, Kaim J, Jertborn M. Parallel analysis of mucosally derived B- and T-cell responses to an oral typhoid vaccine using simplified methods. Vaccine. 2009 Jul 16;27(33):4529-36. doi: 10.1016/j.vaccine.2009.05.005. Epub 2009 May 27. PMID: 19446596.

5 Attridge SR, Johansson C, Trach DD, et al. Sensitive Microplate Assay for Detection of Bactericidal Antibodies to Vibrio cholerae O139. Clin Diagn Lab Immunol 2002; 9(2):383–7. [42]

**Supplementary file 3.** Pre-existing medical conditions and regular systemic or inhaled medications of study population included in safety and immunogenicity analyses (n= 63).

Past operations, allergies not requiring regular medication or the need for oral contraception or postmenopausal hormone replacement therapy were recorded but not reported here as pre-existing medical conditions. Medications taken if necessary were recorded but not reported here.

(n): number of volunteers reporting the specified medical condition or medication

**Pre-existing medical conditions**

Neurologic: migraine (8), restless legs syndrome (1), Ménière’s disease (1), narcolepsy (1)

Psychiatric: depression (5), anxiety disorder (2), attention-deficit/hyperactivity disorder (2), autism (1)

Cardiometabolic: hypertension (5), dyslipidemia (3), diabetes mellitus type II (2), thromboembolism (1), mitral valve prolapse (1)

Respiratory: asthma (8)

Dermatologic: atopy (4), acne (2), allergic dermatitis (1), perioral dermatitis (1), lichen ruber planus (1), fungal nail infection (1)

Gastrointestinal: irritable bowel syndrome (2), functional diarrhoea (1), oesophageal reflux (1), eradicated hepatitis C without cirrhosis (1)

Other: allergy requiring regular antihistamine (5), arthrosis (1), hypothyroidism (1), vasomotor rhinitis (1)

**Regular systemic or inhaled medications**

Cardiometabolic medications: antihypertensive (7), statin (3), diabetes/obesity medication (3), novel anticoagulant (1)

Neurologic and psychiatric medications: antidepressant (6), stimulant (2), dopamine agonist (1), central nervous system depressant (1)

Asthma and allergy medications: antihistamine (5), inhaled cortisone (3), leukotriene receptor antagonist (1)

Other medications: proton pump inhibitor (2) and terbinafine, levothyroxine, nonsteroidal anti-inflammatory drug, retinoid and chondroitin sulphate (1 each).

**Supplementary file 4.** Volunteers reporting adverse events (AEs) probably or possibly related to study vaccines during the one-month study period.

|  | **Group Ch** n= **21**/23^a^  n (%) | **Group Ch+Ty** n= **21**/21^a^  n (%) | **Group Ty** n= **21**/21^a^  n (%) | p-value^b^ |
| --- | --- | --- | --- | --- |
| **All AEs** | 14 (66.7%) | 18 (85.7%) | 15 (71.4%) | 0.440 |
| **Solicited AEs** | 14 (66.7%) | 12 (57.1%) | 13 (61.9%) | 0.946 |
| **Gastrointestinal** | 12 (57.1%) | 10 (47.6%) | 12 (57.1%) | 0.856 |
| Abdominal pain | 7 (33.3%) | 4 (19%) | 7 (33.3%) | 0.543 |
| Loose stools^c^ | 6 (28.6%) | 3 (14.3%) | 5 (23.8%) | 0.645 |
| Diarrhoea^d^ | 0 | 0 | 2 (9.5%) | 0.323 |
| Constipation^e^ | 1 (4.8%) | 2 (9.5%) | 1 (4.8%) | 1.000 |
| Nausea | 3 (14.3%) | 3 (14.3%) | 2 (9.5%) | 1.000 |
| Vomiting | 0 | 0 | 0 | - |
| **Other** | 10 (47.6%) | 9 (42.9%) | 7 (33.3%) | 0.730 |
| Headache | 6 (28.6%) | 5 (23.8%) | 6 (28.6%) | 1.000 |
| Muscle ache/joint pain | 3 (14.3%) | 2 (9.5%) | 2 (9.5%) | 1.000 |
| Temperature >37.5⁰C^f^ | 0 | 0 | 0 | - |
| Chills^f^ | 1 (4.8%) | 1 (4.8%) | 0 | 1.000 |
| Fatigue | 6 (28.6%) | 3 (14.3%) | 1 (4.8%) | 0.132 |
| Rash | 0 | 0 | 0 | - |
| Vertigo | 1 (4.8%) | 1 (4.8%) | 0 | 1.000 |
| **Unsolicited AEs** | 6 (28.6%) | 13 (61.9%) | 11 (52.4%) | 0.083 |
| **Gastrointestinal** | 6 (28.6%) | 12 (57.1%) | 11 (52.4%) | 0.156 |
| Flatulence | 4 (19%) | 5 (23.8%) | 3 (14.3%) | 0.920 |
| Bubbly gut | 2 (9.5%) | 2 (9.5%) | 1 (4.8%) | 1.000 |
| Bloating | 1 (4.8%) | 3 (14.3%) | 3 (14.3%) | 0.682 |
| Belching | 1 (4.8%) | 1 (4.8%) | 1 (4.8%) | 1.000 |
| Heartburn | 1 (4.8%) | 1 (4.8%) | 2 (9.5%) | 1.000 |
| Other gi^g^ | 2 (9.5%) | 2 (9.5%) | 1 (4.8%) | 1.000 |
| **Other**^h^ | 1 (4.8%) | 4 (19%) | 0 | 0.116 |

Volunteers (n= 65) received either oral killed whole-cell recombinant cholera toxin B-subunit vaccine (Group Ch), live *Salmonella* Typhi Ty21a vaccine (Group Ty), or both (Group Ch+Ty, first doses ingested simultaneously).

^a^ Participants with available safety data/Vaccinated participants. Two in Group Ch were lost to follow-up before the second dose of WC-rCTB. Final safety population in bold (n=63).

^b^ P-values were calculated by Fisher’s exact test.

^c^ Loose stools= Participant’s subjective opinion that his/her stools were significantly looser than usual

^d^ Diarrhea= Three or more grade III-V stools/24h (Stool grading: Grade I = Firm; grade II = Soft, formed; grade III = soft, nearly liquid; Grade IV = colourful liquid and Grade V = clear, almost colourless liquid)

^e^ Constipation= participant’s subjective opinion that his/her bowel movements were significantly less frequent and stools more difficult to pass than usual

^f^ Temperature >37.5⁰C was the original solicited AE. Participants were advised to measure the temperature only in case they felt feverish. Only four participants reported chills possibly or probably related to study vaccine and none of them temperature >37.5⁰C

^g^ Other unsolicited gastrointestinal AEs probably or possibly related to study vaccines: In Group Ch one participant reported change of stool consistency from watery to hard and anal irritation, another loss of appetite. In Group Ch+Ty one loss of appetite and one increased appetite. In Group Ty one increased appetite.

^h^ other unsolicited AEs probably or possibly related to study vaccines: one participant in group Ch reported night sweats, insomnia and tinnitus. Four participants in group Ch+Ty reported one of the following: mucosal tenderness of the mouth, runny nose, pruritus and exacerbation of acne.

n, number of participants; %= proportion of participants (n) in respective group

.

**Supplementary file 5.** Severity of adverse event episodes probably or possibly related to study vaccines reported during the one-month study period.

|  | **Group Ch** n=**21**/23^a^  Ep (%) | **Group Ch+Ty** n=**21**/21^a^  Ep (%) | **Group Ty** n=**21**/21^a^  Ep (%) | p-value^b^ |
| --- | --- | --- | --- | --- |
| **All AE episodes** | 59 | 56 | 44 | 0.354 |
| Mild | 44 (74.6%) | 48 (85.7%) | 38 (86.4%) |  |
| Moderate^c^ | 15 (25.4%) | 7 (12.5%) | 5 (11.4%) |  |
| Severe^d^ | 0 | 1 (1.8%) | 1 (2.3%) |  |

Volunteers (n= 65) received either oral killed whole-cell recombinant cholera toxin B-subunit vaccine (Group Ch), live *Salmonella* Typhi Ty21a vaccine (Group Ty), or both (Group Ch+Ty, first doses ingested simultaneously).

^a^ Participants with available safety data/Vaccinated participants. Two in Group Ch were lost to follow-up before the second dose of WC-rCTB. Final safety population in bold (n=63). A total of 14/21 volunteers reported AE episodes in Group Ch, 18/21 in Group Ch+Ty and 15/21 in Group Ty.

^b^ The comparison between groups in severity of AEs was done with ordinal logistic regression using generalized estimating equations. Episodes including AEs were included in analysis.

^c^ Moderate AEs comprised the following episodes: In group Ch 12/15 solicited AEs (headache 6, muscle ache/joint pain 3, fatigue 2 and abdominal pain 1) and 3/15 unsolicited AEs (heartburn 1, flatulence 1 and other 1). In group Ch+Ty 4/7 solicited AEs (headache 2, muscle ache/joint pain 1 and fatigue 1) and 3/7 unsolicited AEs (heartburn 1 and other 2). In group Ty 3/5 solicited AEs (headache 1 and abdominal pain 2) and 2/5 unsolicited AEs (flatulence 1 and bloating 1).

^d^ Severe AE episodes comprised one nausea in Group Ch+Ty and one headache in Group Ty.

Ep, number of adverse event episodes; %= proportion of episodes (Ep) of all episodes in respective group

**Severity grading of adverse events**

| **Mild** | **Moderate** | **Severe** |
| --- | --- | --- |
| An adverse event which is relatively mild and transient in nature, but can be an annoyance although it *does not interfere with normal activities.* | An adverse event which may be uncomfortable but is not hazardous to health. It may be sufficiently discomforting to *interfere with normal activities but does not completely prevent them.* | An adverse event which is *incapacitating and prevents normal activities and/or might pose a hazard to the participant* |

**Supplementary file 6.** Volunteers reporting adverse events (AEs) unlikely related to study vaccines during the one-month study period (both solicited and unsolicited).

|  | **Group Ch** n=**21**/23^a^  n (%) | **Group Ch+Ty** n=**21**/21^a^  n (%) | **Group Ty** n=**21**/21^a^  n (%) | p-value^b^ |
| --- | --- | --- | --- | --- |
| **All AEs** | 8 (38.1%) | 5 (23.8%) | 5 (23.8%) | 0.641 |
| Infection | 5 (23.8%) | 4 (19%) | 5 (23.8%) | 1.000 |
| Respiratory | 4 (19%) | 4 (19%) | 4 (19%) | 1.000 |
| Other infection^c^ | 1 (4.8%) | 0 | 2 (9.5%) | 0.767 |
| Other^d^ | 4 (19%) | 1 (4.8%) | 0 | 0.116 |

Volunteers (n= 65) received either oral killed whole-cell recombinant cholera toxin B-subunit vaccine (Group Ch), live *Salmonella* Typhi Ty21a vaccine (Group Ty), or both (Group Ch+Ty, first doses ingested simultaneously).

^a^ Participants with available safety data/Vaccinated participants. Two in Group Ch were lost to follow-up before the second dose of WC-rCTB. Final safety population in bold (n=63).

^b^ P-values were calculated by Fisher’s exact test

^c^ One gastroenteritis in group Ch and two urinary tract infections in group Ty

^d^ Belching and stomach ache after overeating, neck pain after exercise, functional diarrhea reactivation and delayed period in group Ch and migraine after a night shift in group Ch+Ty, all occurring after Day 14.

n, number of participants; %= proportion of participants (n) in respective group
